# Isolation and characterization of a *Bacillus subtilis* strain that degrades endosulfan and endosulfan sulfate

**DOI:** 10.1007/s13205-013-0176-7

**Published:** 2013-10-01

**Authors:** Ajit Kumar, Narain Bhoot, I. Soni, P. J. John

**Affiliations:** 1 Environment Toxicology Unit, Centre for Advanced Studies in Zoology, University of Rajasthan, Jaipur, 302004 India; 2 Present Address: Centre for Bioinformatics, M.D. University, Rohtak, 124001 India

**Keywords:** Biodegradation, Endosulfan, *Bacillus subtilis*, Characterization

## Abstract

Endosulfan has emerged as a major environmental menace worldwide due to  extensive usage and environmental persistence, seeking its remedial by a cheaper and efficient means. Therefore, natural resource (soil) was explored to search a potential candidate for biodegradation of endosulfan. A soil bacterium was enriched and isolated by applying a strong nutritional selection pressure, using a non-sulfur medium supplemented with endosulfan as sole source sulfur. The microbial strain was found to degrade endosulfan as well as its equally toxic metabolite endosulfan sulfate to non-toxic metabolites (endodiol and endosulfan lactone) very efficiently (up to 94.2 %) within 7 days, estimated qualitatively by thin layer chromatography and quantitatively by gas chromatography-electron capture detection methods. The isolate was characterized for its morphological, physiological, biochemical and 16S rRNA sequencing and identified as a new strain of *Bacillus subtilis* with strain designation AKPJ04, which was deposited with accession number Microbial Type Culture Collection and Gene Bank (MTCC) 8561, at MTCC, Institute of Microbial Technology, Chandigarh, India. The partial 16S rRNA sequence was submitted to Genbank, Maryland, USA, with the accession number EU 258611. The primary investigation for endosulfan degrading gene(s) localization suggested its location on chromosomal DNA.

**Electronic supplementary material:**

The online version of this article (doi:10.1007/s13205-013-0176-7) contains supplementary material, which is available to authorized users.

## Introduction

With the advent of Green Revolution, use of synthetic fertilizers and pesticides has increased at an uncontrolled pace to meet the demands of ever-growing human population. To the darker side of the same, these compounds, particularly, pesticides have posed a serious ecological threat and therefore needs an early scientific attention. Presently, endosulfan is one of the extensively used organochlorine pesticides after the worldwide ban on DDT and BHC. Since then, use of endosulfan has increased dramatically in last three decades. It is used extensively worldwide as a contact and stomach insecticide for Colorado potato beetle, flea beetle, cabbageworm, peach tree borer, tarnished plant bug and as an acaricide on field crop like cotton, paddy, sorghum, oilseeds and coffee (Lee et al. 1995; Kullman and Matsumura 1996; USEPA 2002), apart from its use in vector-control (tsetse fly), as a wood preservative and for the control of home garden pests (C.N.R.C 1975). It is a highly toxic substance and is classified as a Category 1b (highly hazardous) pesticide by USEPA (2002).

There have been several reports of acute poisoning and chronic toxicity of endosulfan. Acute toxicity includes stimulation of Central Nervous System as the major characteristic and is indistinguishable from symptoms of other cyclodienes (USDHHS 1990). Endosulfan has been reported to be highly toxic to aquatic fauna like fish and invertebrates (Sunderam et al. 1992). In addition, there are reported implications in mammalian gonadal toxicity (Sinha et al. 1997), genotoxicity (Chaudhari et al. 1999), teratogenic effects (Yadav 2003) and mutagenic effects (USDHHS 1990). These acute and chronic toxicity and environmental concerns have attracted scientists for an effective and economically viable option search for endosulfan degradation.

Bioremediation has evolved as a very economical and viable process for detoxification of xenobiotics in general and pesticides in particular, as an alternative to existing methods such as incineration and landfill (Gavrilescu and Chisti 2005). Therefore, present investigations were carried out in our laboratory to enrich and isolate endosulfan degrading microorganism from the natural resource (soil), having the past history of endosulfan usage. The isolated strains were studied for their comparative pesticide degradation ability to select the best biodegrader. The best degrading isolate N2 was found to degrade both the isomers of endosulfan (α- and β-isomers) along with the equally toxic metabolite endosulfan sulfate up to 94 % within 7 days (Kumar et al. 2012).

The present report describes the biochemical and molecular characterization and identification of the best endosulfan degrading isolate N2 and comparative analysis of its degradation profile with the standard microorganisms (MOs) reported earlier for endosulfan degradation, *Phanerochaete chrysosporium* (Kullman and Matsumura 1996) and *Mucor thermohyalospora* (Shetty et al. 2000).

## Materials and methods

**Fig. 1:**
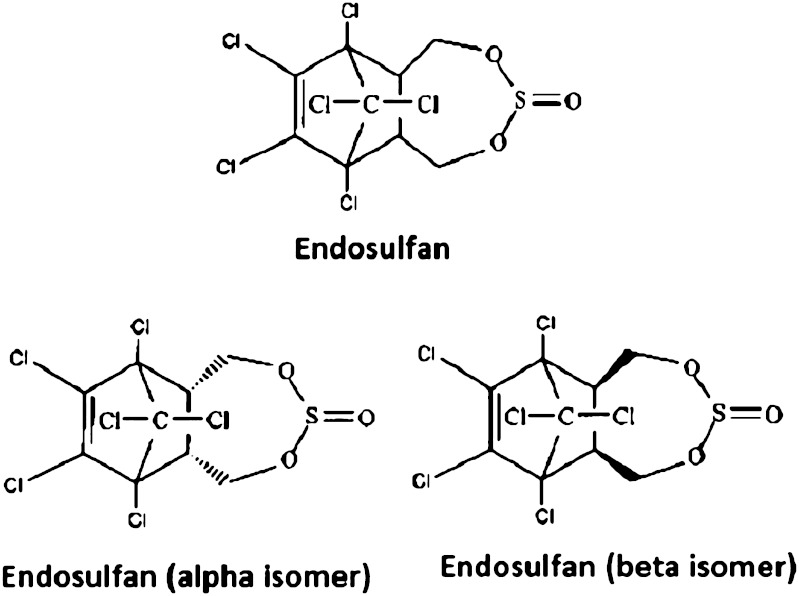
Endosulfan and its isomers (technical grade composition—α: β::7:3)

Technical grade endosulfan (99 % pure), an organochlorine insecticide with the chemical name 6,7,8,9,10,10-hexachloro-1,5,5a,6,9,9a-hexahydro-6,9-methano-2,4,3benzo-dioxathiepin-3-oxide for the study, was gifted by Excel India Pvt. Ltd., Ahmedabad, India (Fig. 1). The endosulfan isomers (α- and β-isomers), endosulfan sulfate and other endosulfan metabolite standards for chromatographic analyses were purchased from Hewlett Packard Company, Wilmington, Delaware, USA. Chloroform, *n*-hexane and acetone of chromatographic grade were used for chromatographic studies. All other chemicals used for the study were of analytical grade. The earlier reported endosulfan degraders, *P. chrysosporium* MTCC 4955 (PC) and *M. thermohyalospora* MTCC 1384 (MT), were selected as standards for the comparative study and were purchased from Microbial Type Culture Collection and Gene Bank (MTCC), Institute of Microbial Technology (IMTECH), Chandigarh.

### Culturing of isolate N2 and standard MOs

The isolate N2 for the study of endosulfan degradation profile was cultured in non-sulfur medium (NSM) supplemented with 50-ppm technical endosulfan as sole source of sulfur (Table 1). The other culture conditions included pH 6.5 with rotational agitation of 130 rpm, and incubation temperature and time of 30 °C and 15 days, respectively. The culture conditions were as per optimized parameters reported earlier, with little modification (Kumar et al. 2012). The other two MOs, PC and MT, were cultured as per their corresponding reports (Kullman and Matsumura 1996; Shetty et al. 2000) for comparative study of endosulfan degradation.

**Table 1:** Composition of (a) non-sulfur medium (NSM) (pH = 6.5), (b) Trace elements solution

S. no.	Chemical	Amount (g/liter)
a		
1	K_2_HPO_4_	0.225
2	KH_2_PO_4_	0.225
3	NH_4_Cl	0.225
4	MgCl_2_·6H_2_O	0.845
5	CaCO_3_	0.005
6	FeCl_2_·4H_2_O	0.005
7	d-Glucose	1.000
8	Trace element solution	1 mg/lt
b		
1	MnCl_2_·4H_2_O	198
2	ZnCl_2_	136
3	CuCl_2_·2H_2_O	171
4	CoCl_2_·6H_2_O	24
5	NiCl_2_·6H_2_O	24

### Analysis of endosulfan degradation and characterization of metabolites

For endosulfan and its metabolites extraction and analysis, 5 ml of cultured broth of the isolate N2, PC and MT were subjected with equal volumes of ethyl acetate and the organic phase was passed through a 6-cm MgSO_4_ column in a Pasteur pipette to remove any residual water. The columns were pre-washed with ethyl acetate. The extracted elutes containing pesticide and metabolites were gently evaporated at 50 °C in oven and were dissolved in acetone (chromatographic grade) and stored in glass vial at 4 °C for further analysis. A fortification test for recovery of endosulfan isomers was carried out for further chromatographic analysis.

The sample extracts of the N2-culture medium were analyzed for the residues of endosulfan by Gas Chromatography-Electron Capture Detection (GC-ECD) method. The analysis was carried out on a Shimadzu Model 2010 Gas chromatograph (GC) equipped with 63Ni Electron capture detector (ECD), and a capillary column HP ultra 2 (US 4293415) 0.52 × 25 × 0.32. The instrument was supported by Lab Solutions software for the analysis of endosulfan (α- and β-isomers) and endosulfan sulfate. The stock standards (200-ppm) of endosulfan isomers and endosulfan sulfate were obtained from Hewlett Packard Company, USA. Stock standards of 100-ppm were prepared by diluting standard mixture in 1:1 solvent mixture of HPLC grade iso-octane and toluene. These stocks were stored under freezing conditions. Working standard of the mixture was prepared from 100-ppm stock solution. 0.5–1.0 ppm of this mixture of endosulfan isomers and endosulfan sulfate was used for calibrating the GC for analyzing residues of endosulfan in the sample analyzed.

For thin layer chromatography (TLC), the dried endosulfan isomers and metabolites, after extraction from the culture, were dissolved in chromatographic grade acetone and applied to neutral Silica gel TLC plates (pre-activated at 80 °C for 30 min). The plates were developed in hexane: chloroform: acetone (9:3:1). The chlorine-containing constituents were visualized by spraying plates with AgNO_3_-saturated methanol and then exposing them to UV-light (Kovacs 1965).

### Characterization and identification of isolate N2

The isolate N2 was subjected to different morphological characterizations (colony morphology, negative staining, gram staining and endospore staining) along with different biochemical characterizations (Cappuccino and Sherman 2005). A growth curve was plotted using NSM with endosulfan as sole source of sulfur for isolate N2 under optimized culture conditions. Its growth was monitored for a temperature range of 15–50 °C, a pH range of 4.0–9.0 and salinity range of 2.0 % NaCl–10.0 % NaCl concentrations for further physiological characterization.

The partial 16S rRNA gene sequencing of isolate N2 was carried out by the custom services of IMTECH, Chandigarh, India, and was subjected to sequence homology search using BLAST (Altshcul et al. 1990) to identify the isolate. The phylogenetic analysis of the isolate N2 was also carried out using CLUSTAL-W (Larkin et al. 2007) for multiple sequence alignment, and a phylogenetic tree was constructed using Neighbour-Joining method of Phylip version 3.69 (Felsenstein 2005).

### Primary localization of endosulfan degrading gene in isolate N2

The isolate N2 was also looked for plasmid content by attempting plasmid isolation by alkaline lysis method (Sambrook et al. 1989) and boiling lysozyme preparation method (Ausubel et al. 2005). *E. coli* DH5α (plasmid strain) was run as positive control in both the plasmid isolation protocol and 1.0 % agarose gel was run for visualization of DNA bands.

## Results and discussion

### Fortification test for endosulfan recovery from microbial cultures

The fortification tests for recovery of endosulfan isomers from microbial cultures during degradation analysis were conducted for five concentrations (0.5, 5.0, 10, 50 and 100 ppm) of endosulfan in the culture broth. The recovery was observed to range from 92.5 to 102.8 % for α-endosulfan and 93.2 to 104.1 % for β-endosulfan with the coefficient of variation ranging from 0.8 to 2.8 % and 0.84 to 4.2 % for α-endosulfan and β-endosulfan, respectively. Based on the fortification analysis of recovery experiments for both endosulfan isomers, the extractions were considered appropriate of residue analysis.

### GC-ECD & TLC analysis of endosulfan degradation profile

After GC-separation and ECD analysis, it was found that 8.65 ppm of α-endosulfan, 5.85 ppm of β-endosulfan and 2.98 ppm of endosulfan sulfate remained in the N2-culture system, accounting for about 71.0 % degradation after 3 days of incubation, as compared to 24.82 ppm of α-endosulfan, 15.81 ppm of β-endosulfan and 6.42 ppm of endosulfan sulfate detected in the control sample accounting for 18.74 % abiological degradation. After 7 days of incubation, the degradation of endosulfan in N2-culture system was found to be 94.0 % with 1.79 ppm of α-endosulfan, 1.21 ppm of β-endosulfan and 0.32 ppm of endosulfan sulfate detected by GC-ECD as compared to 23.93 ppm of α-endosulfan, 14.78 ppm of β-endosulfan and 6.98 ppm of endosulfan sulfate detected in the control sample that accounts for 22.58 % abiological degradation (Table 2).

**Table 2:** Gas chromatography–ECD data of endosulfan isomers and endosulfan sulfate after degradation by isolate N2

Sample	Peak#	Retention time (min)	Compound	Concentration (ppm)	Endosulfan degradation (%)
Control (Day 3)	24	13.818	α-Endosulfan	24.82	18.74
	32	15.751	β-Endosulfan	15.81	
	37	17.207	Endosulfan sulfate	6.42	
Control (Day 7)	25	13.800	α-Endosulfan	23.93	22.58
	32	15.729	β-Endosulfan	14.78	
	37	17.205	Endosulfan sulfate	6.98	
N2 (Day 3)	26	13.757	α-Endosulfan	8.65	71.0
	32	15.708	β-Endosulfan	5.85	
	38	17.207	Endosulfan sulfate	2.98	
N2 (Day 7)	27	13.709	α-Endosulfan	1.79	94.0
	32	15.697	β-Endosulfan	1.21	
	33	17.207	Endosulfan sulfate	0.32	

Culture condition: Media, NSM with 50-ppm technical endosulfan as sole source of sulfur; pH, 6.5; Temperature, 30 °C; Agitation, 130 rpm

Isolate N2 was also investigated for its comparative degradation with positive control cultures of reported standard microbes, namely, *Phanerochaete chrysosporium* (Kullman and Matsumura 1996) and *Mucor thermohyalospora* MTCC 1384 (Shetty et al. 2000). This comparison was made on the basis of analyzing endosulfan degradation and its metabolites’ profile recovered from the respective culture media after 15 days of incubation, under respective standard culture conditions. The TLC-plate analysis revealed six spots, identified as α-endosulfan, β-endosulfan, endosulfan sulfate, endosulfan lactone, endosulfan hydroxyether and endosulfan diol, on the basis of their respective retention factor (*R*_f_) of 0.69, 0.46, 0.36, 0.29, 0.21 and 0.10. Endosulfan sulfate was the only abiotic degradation metabolite observed in the control, while cultures of N2, PC and MT showed endosulfan diol and endosulfan sulfate as the two common metabolites. Endosulfan diol was produced maximum in the cultures of N2, while PC and MT produced the same as minor metabolite. A spot of endosulfan lactone was observed in the cultures of N2, while no such spot was observed for PC and MT. A spot of endosulfan hydroxyether was observed only for PC-culture while no such metabolite was detected in N2 and MT (Table 3).

**Table 3:** TLC profile of endosulfan degradation by isolate N2 and standard cultures of *P. chrysoporium* (PC) and *M. thermohyalospora* (MT)

Metabolites	Rf Value^§^	Control*	N2*	PC	MT
α-Endosulfan	0.69	+++	+	+	+
β-Endosulfan	0.46	+	+	+	+
Endosulfan sulfate	0.36	+	+	+	+
Endosulfan lactone	0.29	–	+	–	–
Endosulfan hydroxyether	0.21	–	–	+	–
Endosulfan diol	0.10	–	+++	+	+

Culture condition: Media, NSM with 50-ppm technical endosulfan as sole source of sulfur; pH, 6.5; Temperature, 30 °C; Agitation, 130 rpm;+++, Formation of major metabolite; +, formation of minor metabolites, –, no metabolite detected

^**§**^ TLC solvent system used: hexane:petroleum ether:acetone (9:3:1)

The major metabolite detected in the isolate N2 was endosulfan diol with little amount of endosulfan lactone. Endosulfan hydroxyether was not detected in the isolate similar to *M. thermohyalospora* MTCC 1384 and in contrast to *P. chrysosporium.* In *P. chrysosporium*, endosulfan diol and endosulfan hydroxyether were detected, while no trace of endosulfan lactone was observed. *M. thermohyalospora* MTCC 1384 had endosulfan diol as the only metabolite detected apart from spots of α-endosulfan, β-endosulfan and endosulfan sulfate, observed commonly in all the samples. These observations were in accordance with their respective reported findings (Kullman and Matsumura 1996; Shetty et al. 2000). Shetty et al. (2000) reported about degradation of endosulfan by *M. thermohyalospora* to form endosulfan diol as the major metabolite. Kullman and Matsumura (1996) found that *P. chrysosporium* degraded endosulfan in endosulfan diol and endosulfan hydroxyether by utilizing both oxidative and hydrolytic pathways for metabolism of this pesticide.

### Morphological characterization of isolate N2

The isolate N2 was found to be Gram +ve, long, rod-shaped bacilli after gram staining and negative staining. It was also found to be a spore former after performing endospore staining with subterminally/centrally positioned, ellipsoidal and no-swollen spores. Isolate N2 was found to form elevated, large, round and convex colonies with shiny mucoid surface as observed from its colony morphology. The size of the bacterium was found to be 2–4 μ in length and <1.0 μ in width. The bacteria were observed to be arranged in singles and pairs.

### Physiological characterization

The isolate N2 was observed to grow in the temperature range of 20–50 °C, while no growth was observed at a temperature of 15 °C (Table 4). Growth of the bacterium was found to occur in the pH range of 6.0–9.0 and a salinity range of 2.0–10.0 % NaCl (Table 4).

**Table 4:** Physiological characterizations of isolate N2

Tests*	Results
Growth at temperatures^#^	
15 °C	−
20 °C	+
30 °C	+
37 °C	+
42 °C	+
50 °C	+
(# pH 6.5, NaCl 2 %)	
Growth at pH	
4.0	−
6.0	+
7.0	+
8.0	+
9.0	+
Growth on NaCl (%)	
2.0	+
5.0	+
7.0	+
10.0	+

−, No growth; +, Growth

* All tests were conducted in nutrient medium

**Fig. 2:**
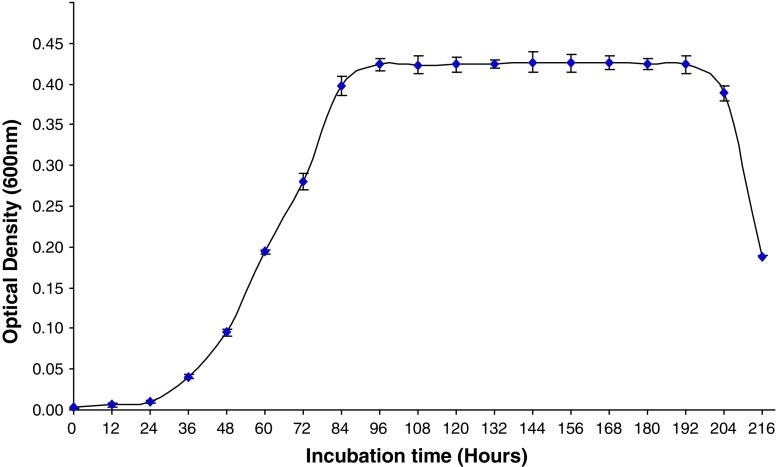
Growth curve of isolate N2. The values are the mean of triplicate samples, and the *bar* indicates the standard error. The culture conditions include the NSM media with 50-ppm technical endosulfan as sole source of sulfur, pH 6.5, incubation temperature of 30 °C and rotator agitation of 130 rpm

The growth curve of N2 was found to be a normal sigmoidal with a delayed lag phase of about 30 h. The log phase was found to extend up to 84 h after which the culture showed stationary phase up to 192 h (8 days). The decline phase initiated after 192 h as evident from growth curve (Fig. 2).

### Biochemical characterization

The isolated bacterium tested positive for citrate utilization, TSI test (acid form glucose), Casein hydrolysis, Esculin hydrolysis, Gelatin hydrolysis, Starch hydrolysis, Nitrate hydrolysis, Catalase test, Lysine decarboxylase test and *ortho*-nitrophenyl glucuronide (ONPG) test. The results were negative for growth on MacConkey agar, Indole test, Methyl red test, Voges–Proskauer tests, H_2_S production, gas from glucase, TSI test (acid from lactose), Urea hydrolysis, Arginine dihydrolase, Ornithine decarboxylase and Phosphatase test.

### Molecular characterization

**Fig. 3:**
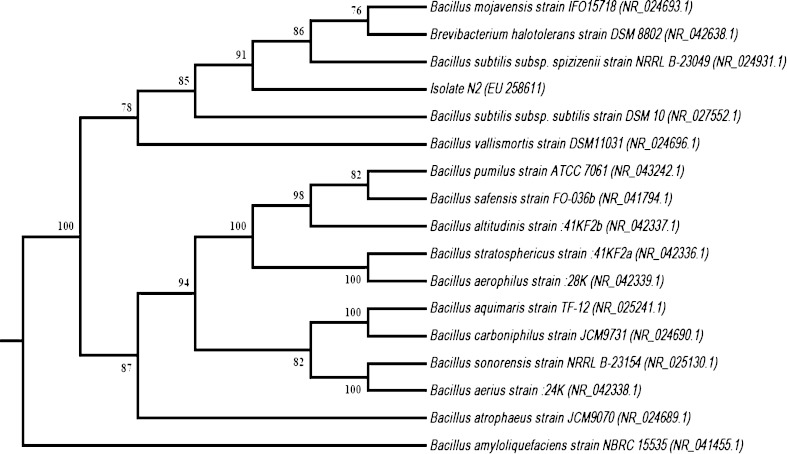
Phylogenetic analysis of isolate N2. The tree is based on 16S rRNA gene sequences from top scoring homologous strains of *Bacillus* and closely related genera. GenBank accession numbers are indicated in the *parenthesis*. *B. Amyloliquefaciens* strains NBRC 15535 was used as the outgroup. Bootstrap values >70 % are given. The scale used for the distance-based tree was 0.01 substitutions per nucleotide position

The partial nucleotide base sequencing (1,396 base pairs) of 16S rRNA gene of isolate N2 was done to identify the bacterium. After performing BLAST search for sequence homology at GenBank, the bacteria showed 100 % identity with *Brevibacterium halotolerans* strain DSM 8802, *Bacillus subtilis* subsp. *subtilis* strain DSM 10, *Bacillus vallismortis* strain DSM11031, *Bacillus mojavensis* strain IFO15718, *Bacillus amyloliquefaciens* strain NBRC 15535 and 99 % identity with *B. subtilis* subsp. *Spizizenii* strain NRRL B-23049 with 100 % query coverage of 16S ribosomal RNA gene. Multiple sequence alignment using CLUSTAL-W was performed for the top scoring sequences of BLAST results showing 95 % and above maximum indent with the query coverage of 97 %. The phylogenetic tree was obtained using .phy output file of CLUSTAL-W with the help of NJ-method of Phylip (Fig. 3).

On the basis of above characterizations, the isolate was identified as a new strain of *B. subtilis* and was deposited at MTCC, IMTECH, Chandigarh, as strain designation AKPJ04 and accession number MTCC 8561, while the 16S rRNA gene sequence of the same was deposited and later published at Genbank with the accession number EU 258611.

The bacterial genus *Bacillus* has also been reported earlier to have endosulfan degradation ability. A bacterial co-culture consisting of two *Bacillus* sp. (MTCC 4444 and MTCC 4445) has been reported to degrade and reduce the toxicity of endosulfan, utilizing the pesticide as carbon source (Awasthi et al. 1997, 2003). The present isolate, *B. subtilis* MTCC 8561, is probably the first *Bacillus* sp. to be reported till date which can utilize endosulfan as sulfur source (Kataoka and Takagi 2013) and degrade it very efficiently up to 94 %. Other bacterial system reported to degrade endosulfan using it as sulfur source are *Alcaligenes faecalis* strain JBW4 (Kong et al. 2013), *Pseudomonas fluorescens* (Giri and Rai 2012), *Achromobacter xylosoxidans* strain C8B (Singh and Singh 2011), *Pseudomonas* and *Burkholderia* (Hussain et al. 2007), *Arthrobacter* (Weir et al. 2006), *Pandoraea* (Siddique et al. 2003), *Mycobacterium* (Sutherland et al. 2000) and *Micrococcus* (Guha et al. 1999). Degradation of endosulfan by bacterial consortia isolated from contaminated soil has also been recently reported (Bhattacharjee et al. 2013).

### Primary localization of endosulfan degrading gene(s) of isolate N2

**Fig. 4:**
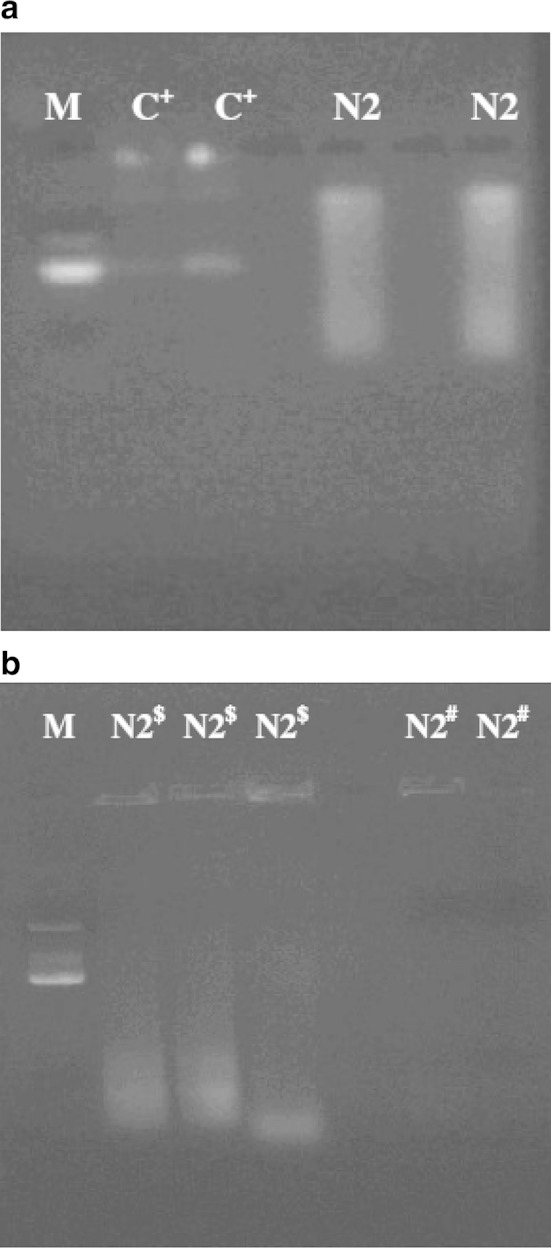
**a** Plasmid isolation from isolate N2. Agarose gel showing plasmid isolation results of isolate N2 by alkaline lysis method. [*M* marker lane, *C+* positive control lane (*E. coli* DH5α), *N2* isolate N2 lane]. **b** Plasmid isolation from isolate N2. Agarose gel showing plasmid isolation results of isolate N2 by lysozyme treatment method. (*M* Marker lane, *N2*^*$*^ isolate N2 lane without RNase treatment, *N2*^*#*^ isolate N2 lane after RNase treatment)

When plasmid isolation from the isolate N2 was attempted by alkaline lysis method, a smear of RNA was obtained when observed under UV-light after agarose gel electrophoresis, while the positive control (*E. coli* DH5α) showed two discrete plasmid bands of closed circular plasmid and nicked plasmids (Fig. 4a). While attempting plasmid isolation, using lysozyme–heat shock treatment method, a smear of RNA was obtained (N2^$^ lane of Figs. 4b). No band was observed under UV-light when the isolated nucleic acid was treated with RNase and electrophoretic run (Lane N2^#^ of Fig. 4b). It may therefore be inferred that isolate N2 does not harbor any plasmid, as there were negative results of plasmid isolation against the positive control. The isolate N2 (*B. subtilis*) was found to be devoid of any plasmid. The plasmid extraction was carried out by alkaline lysis prep method and confirmed by lysozyme boiling—miniprep method. This observation suggests that the endosulfan degrading gene(s) is located on chromosomal DNA. This finding is in accordance with the reports about chromosome-located *esd*-gene of *Mycobacterium* (Sutherland et al. 2002) and *ese*-gene of *Arthrobacter* (Weir et al. 2006), which have been shown responsible for endosulfan degradation.

**Fig. 5:**
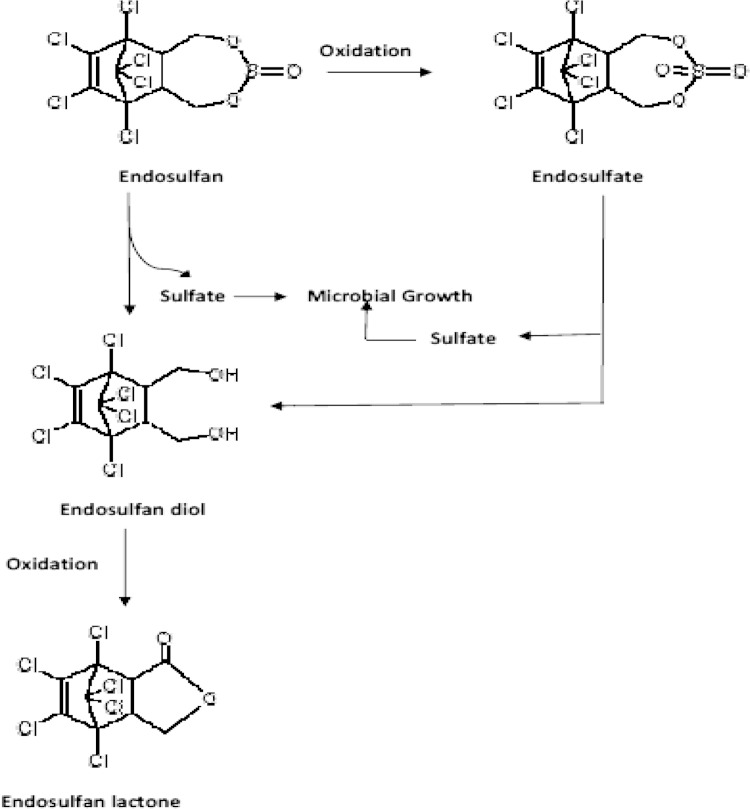
Proposed pathway for metabolism of endosulfan by *Bacillus subtilis* MTCC 8561

## Conclusion

The findings of present investigations suggest that the isolate is probably the first *Bacillus* sp. reported till date, to use endosulfan as sulfur source and the extent of biodegradation of the pesticide is also very high as compared to other reported microbes. The major metabolite detected after degradation by the said isolate (*B. subtilis*) is endosulfan diol along with endosulfan lactone and endosulfan sulfate detected as minor products. From the metabolites detected after degradation, it has been inferred that the bacterium metabolizes endosulfan and its stable but equally toxic, oxidized product, endosulfan sulfate by directly hydrolyzing it to endosulfan diol followed by oxidation to form endosulfan lactone. The high amount of endosulfan diol, detected after bacterial degradation of the pesticide, suggests that the bacterium is hydrolyzing the compounds to release sulfite group from endosulfan and sulfate group from endosulfan sulfate for using them as sulfur source in metabolism and growth. The proposed degradation pathway of endosulfan by *B. subtilis* AKPJ04 is as represented in Fig. 5. Both the major metabolites produced are non-toxic in nature and thus, the isolate holds promise to be a very good candidate for bioremediation of endosulfan. The present study paves a good platform for scaling and validating the results from shake flask level to soil study. The present work also beckons for the identification and characterization of the gene(s) and enzymes, responsible for endosulfan degradation, that are presently under our investigation.

## Electronic supplementary material

Below is the link to the electronic supplementary material.

Supplementary material 1 (DOCX 469 kb)
