# Supplementary material for: Isolation and characterization of a Bacillus subtilis strain that degrades endosulfan and endosulfan sulfate
Source: 3 Biotech. 2013 Oct 1;4(5):467–75. doi: 10.1007/s13205-013-0176-7 (PMC4162894; doi:10.1007/s13205-013-0176-7)
Supplement: Supplementary file 1 — Supplementary material 1 (DOCX 469 kb) [file 13205_2013_176_MOESM1_ESM.docx]

**
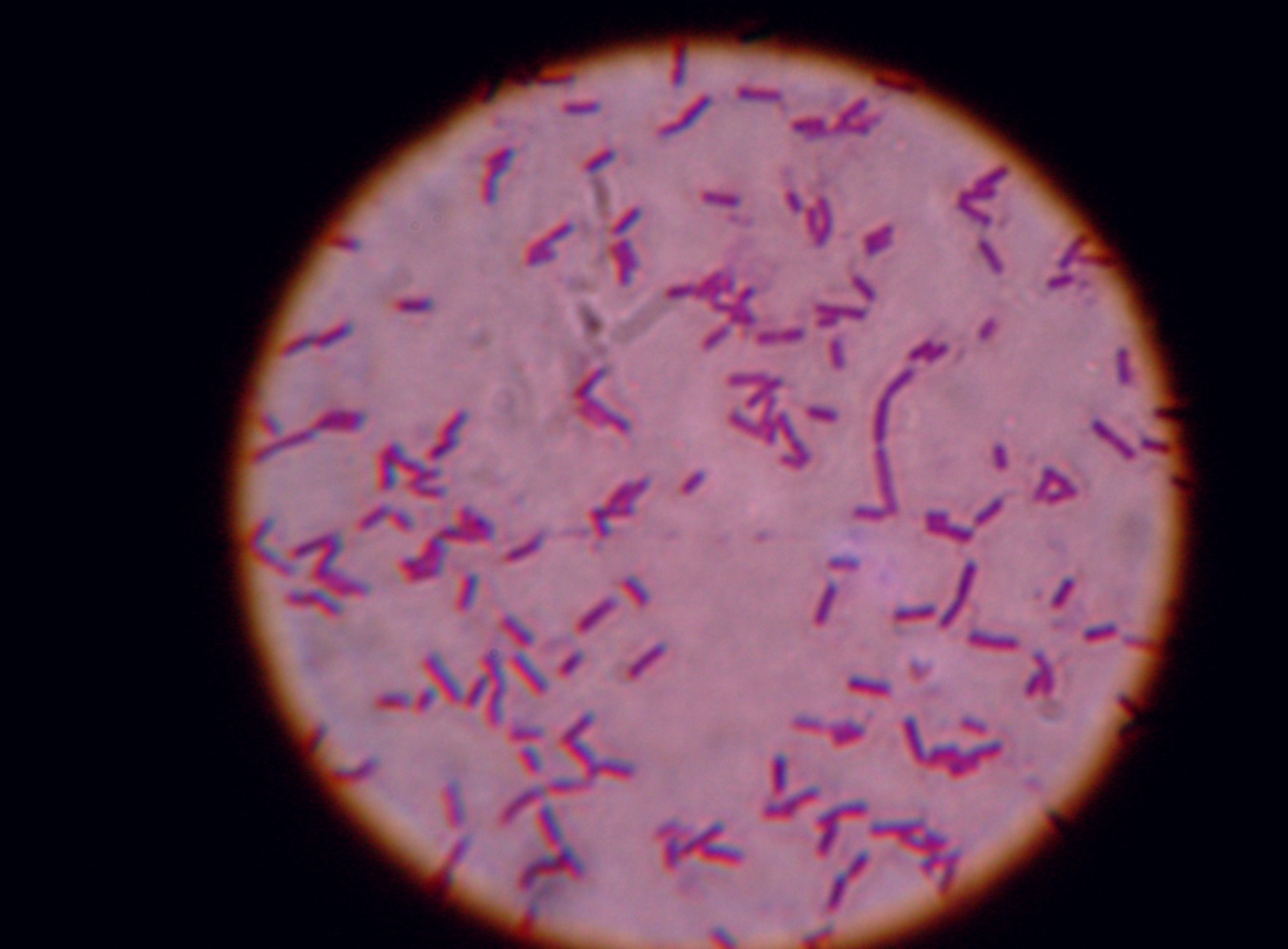
**

**Figure 1Suppl.:** Gram staining. The isolate N2 morphology is figured out to be Gram positive, rod shaped with some dividing cells also visible.

**
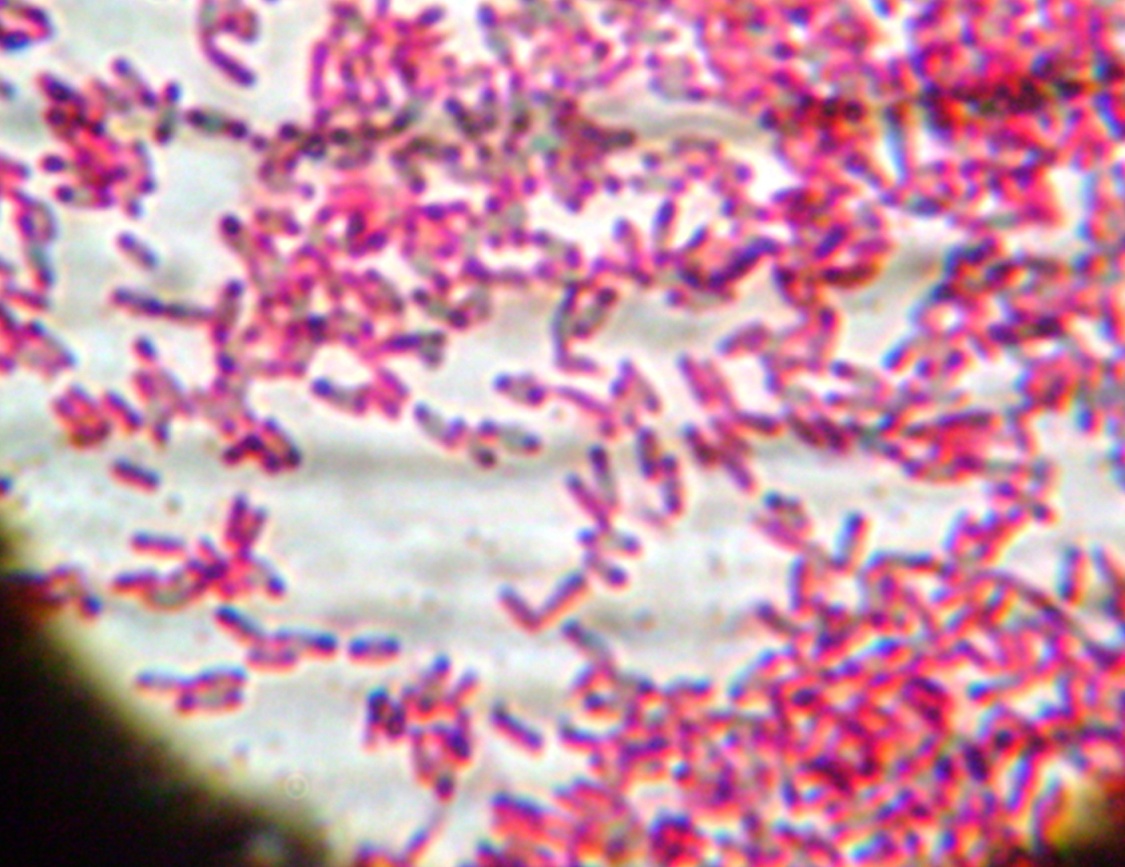
**

**Figure 2Suppl.:** Endospore staining. The isolate N2 appears to form endospore with sub-terminally/centrally positioned, ellipsoidal and no-swollen spores

**Table 1Suppl.-** Biochemical characterizations of isolate N2

| Tests | Results |
| --- | --- |
| Growth on MacConkey agar Indole test  Methyl red test  Voges Proskauer test  Citrate utilization  H_2_S production  Gas from glucose  Acid from glucose (TSI test)  Acid from lactose (TSI test)  Casein hydrolysis  Esculin hydrolysis  Gelatin hydrolysis  Starch hydrolysis  Urea hydrolysis  Nitrate reduction  Catalase test  Oxidase test  Lysine decarboxylase  Arginine dihydrolase  Ornithine decarboxylase  Phosphatase test  ONPG test | -  -  -  -  +  -  -  +  -  +  +  +  +  -  +  +  W  +  -  -  -  + |

**-**: No growth **+**: Growth **W**: Weak growth
